# Molecular Taxonomic Profiling of Bacterial Communities in a Gilthead Seabream (*Sparus aurata*) Hatchery

**DOI:** 10.3389/fmicb.2017.00204

**Published:** 2017-02-14

**Authors:** Gianmaria Califano, Sara Castanho, Florbela Soares, Laura Ribeiro, Cymon J. Cox, Leonardo Mata, Rodrigo Costa

**Affiliations:** ^1^Microbial Ecology and Evolution Research Group, Centre of Marine Sciences, University of AlgarveFaro, Portugal; ^2^Institute for Inorganic and Analytical Chemistry, Friedrich-Schiller-Universität JenaJena, Germany; ^3^Portuguese Institute for the Ocean and Atmosphere, Aquaculture Research StationOlhão, Portugal; ^4^Plant Systematics and Bioinformatics, Centre of Marine Sciences, University of AlgarveFaro, Portugal; ^5^MACRO—the Centre for Macroalgal Resources and Biotechnology, James Cook UniversityTownsville, QLD, Australia; ^6^Department of Bioengineering, Institute for Bioengineering and Biosciences, Instituto Superior Técnico, Universidade de LisboaLisbon, Portugal

**Keywords:** aquaculture, bacterial diversity, fish microbiome, host-microbe interactions, bacterial pathogens

## Abstract

As wild fish stocks decline worldwide, land-based fish rearing is likely to be of increasing relevance to feeding future human generations. Little is known about the structure and role of microbial communities in fish aquaculture, particularly at larval developmental stages where the fish microbiome develops and host animals are most susceptible to disease. We employed next-generation sequencing (NGS) of 16S rRNA gene reads amplified from total community DNA to reveal the structure of bacterial communities in a gilthead seabream (*Sparus aurata*) larviculture system. Early- (2 days after hatching) and late-stage (34 days after hatching) fish larvae presented remarkably divergent bacterial consortia, with the genera *Pseudoalteromonas, Marinomonas, Acinetobacter*, and *Acidocella* (besides several unclassified *Alphaproteobacteria*) dominating the former, and *Actinobacillus, Streptococcus, Massilia, Paracoccus, and Pseudomonas* being prevalent in the latter. A significant reduction in rearing-water bacterial diversity was observed during the larviculture trial, characterized by higher abundance of the *Cryomorphaceae* family (*Bacteroidetes*), known to populate microniches with high organic load, in late-stage rearing water in comparison with early-stage rearing-water. Furthermore, we observed the recruitment, into host tissues, of several bacterial phylotypes—including putative pathogens as well as mutualists—that were detected at negligible densities in rearing-water or in the live feed (i.e., rotifers and artemia). These results suggest that, besides host-driven selective forces, both the live feed and the surrounding rearing environment contribute to shaping the microbiome of farmed gilthead sea-bream larvae, and that a differential establishment of host-associated bacteria takes place during larval development.

## Introduction

Fish farming is the fastest-growing segment within the global agribusiness, with a compound annual growth rate of 9% (FAO, 2014). Currently, worldwide production of farmed fish totals 70.5 million tons per year vs. 93.7 million tons of wild captures. *Sparus aurata* (gilthead seabream) is the third most cultivated marine fish species worldwide, with around 160,000 tons produced every year (FAO, 2012). Most of the gilthead seabream farming takes place in southern European countries (Greece, Italy, Spain, Portugal and France) and Turkey, and the fish is exported, chiefly within the European market, as a highly valuable reared species (FAO, 2012). Even though the efficiency of rearing adults from larvae is increasing, land-based fish larviculture remains a major production bottleneck keeping fish farming output below market expectations. Mortality rates in fish aquaculture during the first 30 days after egg hatching usually range from 80 to even 100% of the initial pool of hatched larvae (Uribe et al., 2011; Vadstein et al., 2013). Besides the typical r-selection strategy of most fish species, characterized by high reproductive recruitment but low survival of the young, high mortality rates observed in fish larviculture are believed to result from greater disease incidence caused by opportunistic/pathogenic bacteria (Olafsen, 2001; Hache and Plante, 2011; Vadstein et al., 2013). This hypothesis has propelled much research on the improvement of fish larvae well-being in aquaculture (Bergh, 2000; Bachère, 2003; Kesarcodi-Watson et al., 2008; Vadstein et al., 2013). Most of the efforts in this regard have focused on the manipulation of existing microbiota and/or enhancing disinfection protocols–e.g., through the use of few culturable, pre- and probiotic bacteria - and on the treatment, cleaning or circulation of the rearing water (Makridis et al., 2005; Sáenz De Rodrigáñez et al., 2009; Conceição et al., 2010; Attramadal et al., 2012). Yet wider exploitation of the entire aquaculture microbiota for improved fish rearing, although desirable, remains difficult owing to our limited view of the identity and activity of the majority of microorganisms that mediate nutrient cycling and disease incidence in land-based fish farming. In fact, the diversity and function of free-living and host-associated microorganisms in fish larviculture has seldom been investigated, constituting a true gap of knowledge not only in aquaculture (Vadstein et al., 2013) but also in fish physiology, developmental and microbiome research. This hinders our understanding of the establishment of microbial communities in early fish developmental stages, and thus a more comprehensive perspective of fish biology in the light of its associated microbiome. Moreover, it also hampers our ability to mitigate the losses presumably caused by harmful microorganisms in intensive fish aquaculture.

The application of next-generation sequencing (NGS) technologies to the study of host-associated microorganisms is spurring significant advances in our understanding of symbiotic relationships and metazoan evolution (Rosenberg et al., 2007; Consortium, 2012; Hentschel et al., 2012; Egan et al., 2013; McFall-Ngai, 2014). Fish microbiome research is currently gaining momentum although it may be considered relatively incipient in comparison with the existing body of knowledge on well-studied hosts, such as humans and plants (Llewellyn et al., 2014). Early molecular surveys based on fingerprinting techniques, such as PCR-DGGE and T-RFLP have enabled a broader characterization of microbial assemblages in farmed fish to be made than previous studies relying on microbial cultivation-dependent methods, revealing the predominance of *Proteobacteria, Firmicutes* and *Bacteroidetes* in the gastrointestinal (GI) tract of fish (Pond et al., 2006; Hovda et al., 2007; Nayak, 2010). Recent NGS assessments of the fish microbiome not only have continued to focus on the GI apparatus (gut, intestine and their contents) of adult fish (Rurangwa et al., 2015; Smith et al., 2015; Schmidt et al., 2016), improving our knowledge of the microbiome of commercially valuable teleost species, such as trout, carp, sturgeon and cod (see Llewellyn et al., 2014 for a comprehensive review). They have also enabled novel insights into the microbiota of fish juveniles (Bakke et al., 2015; Giatsis et al., 2015; Rurangwa et al., 2015).

The first demonstration of bacteria adhering to fish eggs dates back 60 years ago (Oppenheimer, 1955), and the importance of early-stage microbes to fish survival, development and disease susceptibility is well-known (Hansen and Olafsen, 1999; Olafsen, 2001; Vadstein et al., 2013). Recent molecular-based studies have enabled a better circumscription of the bacterial consortia associated with, for instance, cod larvae (Bakke et al., 2013, 2015), but comprehensive knowledge of the structure of microbial communities (especially regarding the identity of their dominant and rare members) relevant to intensive fish larviculture still needs to be determined for a wide range of economically important species. In this study, we used a trans-disciplinary approach, coupling state-of-the-art fish larviculture to NGS taxonomic profiling of bacterial communities, to delineate the autochthonous bacterial consortium of farmed gilthead seabream larvae, and to reveal the participation of exogenous microorganisms in shaping this consortium. We determine bacterial community composition and diversity during a gilthead seabream larval rearing trial using 454 pyrosequencing of 16S rRNA genes amplified from the metagenomes of (1) fish larvae at early [2 days after hatching (DAH)] and late (34 DAH) developmental stages, (2) their live feed, and (3) rearing-water.

## Materials and methods

### Rearing of gilthead seabream larvae

Gilthead seabream larvae were reared at the Aquaculture Research Station (EPPO) of the Portuguese Institute for the Sea and the Atmosphere (IPMA), hereafter termed “EPPO-IPMA,” using methods that were similar to production-scale procedures (Ferreira, 2009). To rear the larvae until their mature stage, an experimentally controlled flow-through system was employed. Water temperature (19.2 ± 1.23°C), salinity (36 ± 1 psu) and dissolved oxygen (7.0 ± 1.05 mg/L) were kept stable throughout the experiment, and light intensity was set at approximately 800 lux within a photoperiod of 14 h light (starting at 9 am) and 10 h dark. Water renewal rate ranged from 20 to 45% per h depending on the type of prey used for feeding (see below), draining through an 80 μm to a 500 μm mesh (Castanho, 2014). The adjacent Ria Formosa lagoon, a highly productive ecosystem well interconnected with coastal seawater, was used as the source of water entering the system.

**Figure 1:**
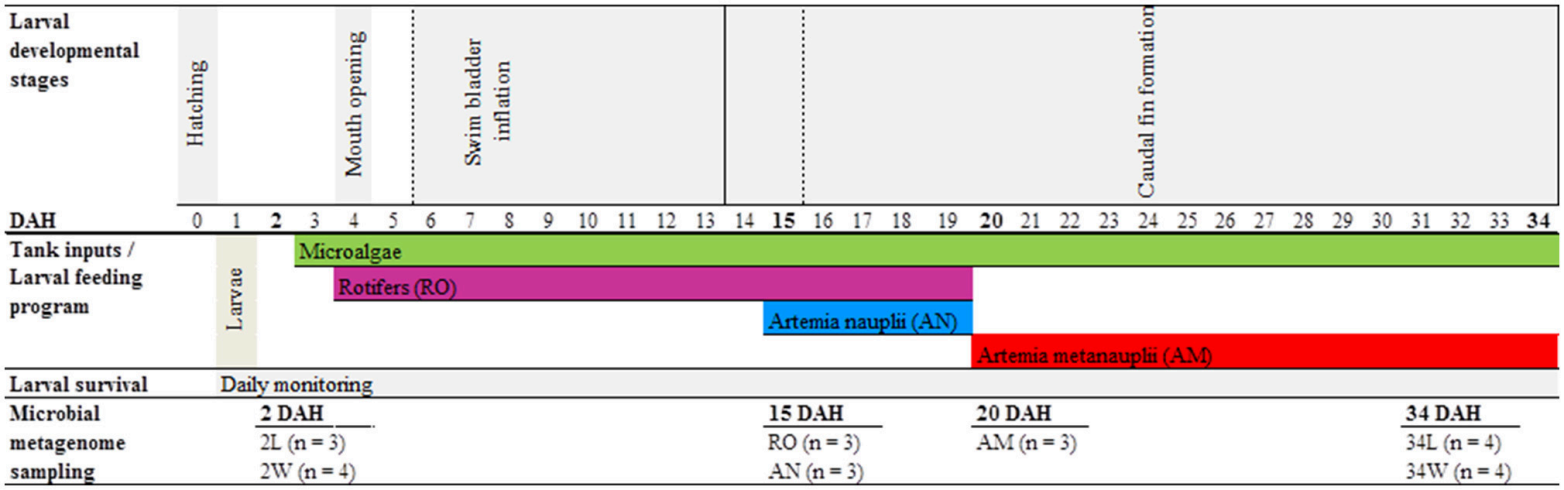
**Experimental design and sampling methodology**. Seven sample categories were used for bacterial community profiling along the rearing trial, as follows. Rearing-water samples taken at 2 (2 W) and 34 (34 W) days after hatching, seabream larvae samples taken at 2 (2 L) and 34 (34 L) days after hatching, rotifers (RO), *Artemia* sp. nauplii (AN) and *Artemia* sp. metanauplii (AM). In “Larval developmental stages”: dashed lines delineate start and end of swim bladder inflation; solid lines delineate start and end of caudal fin formation. In “Microbial metagenome sampling”: in brackets is the number of independent replicates analyzed for each of the sample categories.

Gilthead seabream eggs were obtained from broodstock under captivity at EPPO-IPMA, and incubated at 18 ± 0.5°C in 200 L cylindro-conical fiberglass tanks at a density of 0.5 g.L ^−1^ for 2 days. One day after hatching, fish larvae were distributed across four independent rearing tanks (200 L) at a density of 100 larvae.L ^−1^. Only live feed was provided to the larvae during the entire rearing period, which comprised 35 days from egg hatching to complete organs' development. Larvae were fed rotifers (*Brachionus* spp.) and artemia (*Artemia* sp., nauplii and metanauplii stages) in accordance with larval developmental stage and mouth size (Figure 1, see details below). While rotifers were produced using a batch culture system established at EPPO-IPMA (Ferreira, 2009), artemia nauplii and metanauplii were obtained from Viet Nam Brine Shrimp (VNBS, Golden Lotus Trading LLC, USA) and from Salt Lake Aquafeed (Catvis BV, The Netherlands), respectively. After decapsulation (Ferreira, 2009), artemia cysts were incubated at a density of 4 cysts. mL^−1^ at 27°C and 27 psu under strong aeration. While artemia nauplii were harvested at hatching to be directly used as food, artemia metanauplii were harvested at hatching to be nutritionally enriched prior to larvae provision. Both rotifers and artemia metanauplii were enriched with the commercial product RedPepper® (Bernaqua NV, Belgium) following the supplier's recommendations for each. Prior to feeding, rotifer and artemia pools were washed with flow-through seawater to minimize the input of allochtonous organic material into the tanks. Rotifers were provided to larvae from 4 DAH (when the latter opened their mouth) until 19 DAH. Artemia nauplii were given from 15 DAH until 19 DAH, and metanauplii from 20 DAH until the end of the rearing trial (34 DAH, Figure 1). Live preys were provided *ad libitum*, with a minimum concentration of *c. five* rotifers and one artemia nauplii. mL^−1^ to compensate for the restricted larval mobility at the earliest developmental stages. The “green water technique” (addition of microalgae to the rearing tanks) was applied using a mixture of *Nannochloropsis oculata* (1.2 × 10^5^ cells. mL^−1^) and *Isochrysis* aff. *galbana* (3.0 × 10^4^ cells. mL^−1^) since mouth opening until the end of the trial, totalizing 1.5 × 10^5^ microalgal cells. mL^−1^ added daily to the rearing tanks. Shortly, this technique helps maintaining live feed nutritional profiles. Castanho (2014) performed assessments of larval wellbeing during the course of the experiment, including larval survival, growth, morphology and development. Fish larvae survival (16.5 ± 2.72%) by the end of the experiment (35 DAH) was considered satisfactory (Castanho, 2014).

### Bacterial community profiling: sampling and total community DNA extraction

The sampling scheme used for bacterial community profiling is depicted in Figure 1. Five host- and particle-associated microhabitats were inspected for bacterial community diversity and composition using 454 pyrosequencing of 16S rRNA gene reads amplified from “total community” DNA (TC-DNA) samples. The microhabitats were (1) rearing-water from 2 and 34 DAH, (2) gilthead seabream larvae from 2 and 34 DAH, (3) rotifers, (4) artemia nauplii and (5) artemia metanauplii, totalizing seven hereafter called “sample categories” to include the temporal analysis (2 DAH vs. 34 DAH) of water and larvae microhabitats. Either three or four independent replicate samples were used in the characterization of each sample category (Figure 1).

Rearing-water samples consisted of 2 L volumes taken separately from each of the four experimental tanks at 2 and 34 DAH using disinfected (70% ethanol) plastic beakers, thereby comprising four independent replicates from each sampling point. Rearing-water samples were first passed through a disinfected 150 μM nylon mesh, and then filtered through 0.22 μM pore-sized nitrocellulose filters (Millipore, Billerica, MA, USA) with the aid of a vacuum pump. Filters were stored at −80°C until TC-DNA extraction. Active gilthead seabream larvae were also harvested at 2 and 34 DAH. Each sample consisted of about 0.2 g (wet weight) larval pools, corresponding to approximately 50 larvae from 2 DAH and 6 larvae from 34 DAH, respectively, taken from each of the four experimental rearing tanks using a disinfected 150 μM nylon mesh. Larval pools were gently rinsed three times with sterile artificial seawater to remove microbial cells not firmly attached to the larvae. Larval samples (independent replicates from 2 and 34 DAH, Figure 1) were then transferred to sterile, 2 mL polypropylene tubes and stored at −80°C until TC-DNA extraction. To determine the structure of bacterial communities added each day to the rearing tanks through the provision of feed organisms, three independent replicates of the live feed were sampled (prior to their introduction to the tanks), at random days during the rearing trial (depiction of live feed collection dates was simplified in Figure 1 for the sake of clarity). Live feed replicate samples consisted of 0.2 g pools of each rotifers, artemia nauplii and metanauplii placed into sterile, 2 mL polypropylene tubes after harvesting with disinfected beakers and gentle rinsing (3x) with sterile artificial seawater. Samples were kept at −80°C until TC-DNA extraction.

TC-DNA extraction from all sample categories was carried out using the Ultra Clean® Soil DNA isolation kit (MO BIO Laboratories Inc., Carlsbad, CA, USA). For rearing-water samples, filters obtained as above were first cut into smaller pieces with sterile scissors prior to TC-DNA extraction following the manufacturer's instructions. Larvae, rotifer and artemia (nauplii and metanauplii) samples were first thoroughly homogenized in 500 μL artificial sterile seawater using a 10 cm^3^ Potter-Elvehjem PTFE pestle and glass tube (Scherf-Präzision Europa GmbH, Meiningen, Germany) before being subjected to TC-DNA extraction. Here, an enzymatic lysis step was introduced after mechanical shearing—via bead beating—of the sample material to enable higher DNA yields. This consisted of two successive, 1 h incubation periods with 10 mg/mL lysozyme (Merck-Millipore, Billerica, MA, USA) at 37°C (Pangastuti et al., 2010) and 2 mg/mL proteinase K (Merck-Millipore) at 55°C (Sáenz De Rodrigáñez et al., 2009; Bakke et al., 2013).

### Bacterial community profiling: 454 pyrosequencing and data processing

For bar-coded 454 pyrosequencing, a nested PCR approach was employed to enable standardized 16S rRNA gene amplification from all TC-DNA samples, including 2 DAH fish larvae which presented the lowest DNA yields. In the first PCR, *c*. 10 ng template DNA were used for the amplification of near full-length bacterial 16S rRNA genes using 30 thermal cycles and the universal primer pair F27 (AGAGTTTGATCMTGGCTCAG)—R1492 (TACGGYTACCTTGTTACACTT) (Weisburg et al., 1991), as described elsewhere (Hardoim et al., 2012). The resulting amplicons (2 μL) were used as template in a second PCR with the Ribosomal Database Project (RDP) primer set (V4_titF-AYTGGGYDTAAAGNG and V4_titR-TACNVRRGTHTCTAATYC), which targets the V4 hypervariable region of bacterial 16S rRNA genes, generating amplicons of around 248 bp in length. PCR amplification took place using the PCR Master Mix Kit (QIAGEN GmbH, Hilden, Germany), containing 2.5 units *Taq* DNA polymerase, 1.5 mM MgCl_2_, 1X QIAGEN PCR Buffer and 0.2 mM dNTPs (final concentrations), to which 0.2 μM of each primer were added. Each sample was tagged by different 8-mer barcodes attached to the reverse primer (Appendix S1, Supplementary Material). Thermal cycling involved a touchdown procedure to improve the retrieval of amplicons of the correct size (especially needed for 16S rRNA gene amplification from TC-DNA of 2 DAH larvae), with initial denaturation at 94°C for 4 min, followed by 10 cycles of 30 s at 94°C, 45 s at 65–55°C and 1 min at 68°C. Further 20 cycles followed as described above, except for the use of a constant annealing temperature of 55°C and a final elongation step at 68°C for 10 min. Two 25 μL amplifications were carried out per sample. The final 50 μL amplicon mixtures of each sample were delivered for pyrosequencing on a 454 Genome Sequencer GS FLX Titanium platform (Roche Diagnostics Ltd, West Sussex, UK) at Genoinseq (Biotechnology Innovation Center, Cantanhede, Portugal). For more details on sequencing procedures, see Appendix S1.

Processing and analysis of 454 pyrosequencing data followed the approach and scripts of Hardoim and Costa (2014) and Hardoim et al. (2014), with a few modifications. In summary, raw data were handled with AmpliconNoise (Quince et al., 2011) for the stringent retrieval of high-quality sequences and removal of homopolymers and chimeras. Sequences were subsequently trimmed using Galaxy (http://usegalaxy.org/) to obtain reads between 150 and 260 bp in length. Processing of quality-filtered sequences was performed with the Quantitative Insights Into Microbial Ecology (QIIME) software package (Caporaso et al., 2010). Operational taxonomic units (OTUs) were defined at ≥97% 16S rRNA gene sequence similarity using the UCLUST method (Edgar, 2010). Representative sequences of each OTU were picked using QIIME default parameters, and aligned employing Infernal (Nawrocki et al., 2009) using a STOCKHOLM file of pre-aligned sequences and secondary structures. Taxonomic assignment of representative sequences was carried out with the BLAST taxonomy assigner method using the latest Greengenes database (release 13_05) within the QIIME environment. After OTUs unclassifiable at the domain level or identified as mitochondria and chloroplasts were removed, a final OTU vs. samples table was generated and used for downstream analyses. These comprised (i) estimates of bacterial richness (Chao1) and diversity (Shannon's index) across microhabitats, (ii) phylum- and genus-level bacterial composition in individual and pooled samples per microhabitat, (iii) determination of OTUs specific to and shared by microhabitats using Venn diagrams, and (iv) multivariate analysis of OTU data. The latter was performed via UPGMA clustering and Principal Coordinate Analysis (PCoA) of OTU profiles using both the weighted and unweighted Unifrac metric. Analyses (i) to (iv) were undertaken using two datasets, with and without singleton OTUs, and results from the first dataset are shown unless otherwise stated. We used size-normalized sample libraries to perform analyses (i), (ii), and (iv), whereas the exploration of the full (non-normalized) quality-filtered dataset was employed in Venn Diagram constructions, and to create absolute abundance ranks of OTUs per sample categories and across the whole dataset. The taxonomy of the most differentiating OTUs (see below) was verified and refined, if needed, using the latest SILVA database (version 123.1 of March 29 2016, http://www.arb-silva.de/download/archive/release_123_1) and custom phylogenetic assessments within the software package ARB (Ludwig et al., 2004) as reported elsewhere (Costa et al., 2013; Keller-Costa et al., 2014).

Sequencing data were deposited in the European Nucleotide Archive (ENA) under the study accession number PRJEB9367, with sample accession numbers ERS726185-ERS726201 (host-associated samples) and ERS726303–ERS726310 (rearing-water samples). A sample vs. quality-filtered OTUs table with the corresponding taxonomic assignment of each OTU, including singleton OTUs with verified phylogenetic validity, is provided as Supplementary Material (Table S1, Supplementary Material).

### Statistical analyses

Normality (Shapiro-Wilk) and equal variance tests were performed to inspect the distribution of the OTU richness and diversity measures, as well as of relative abundance values of the most dominant bacterial phyla and genera found across the seven sample categories, all estimated from 454-pyrosequencing data. One-Way Analysis of Variance (ANOVA) was performed on log-transformed alpha diversity measures (OTU richness, Chao1 and Shannon indices), all of which showing normal data distributions, to test whether mean values obtained for all sample groups were equal, followed by all pair-wise multiple comparison procedures using the Holm-Sidak method to determine significance between groups, in our case the seven sample categories. The Kruskal-Wallis test (One-Way ANOVA on Ranks) was employed to test whether the relative abundances of the most dominant bacterial phyla and genera changed significantly across the seven sample categories, given the absence of normal data distributions in most cases. A *post-hoc* Dunn's test was used to verify differences among sample categories in a pair-wise manner. Analyses were conducted using SigmaPlot 11 (Systat Software Inc., London, UK). Jackknifed beta-diversity procedures were run within the QIIME environment (jackknifed_beta_diversity.py) to test the statistical validity of sample groups generated by cluster and ordination (PCoA) analyses of OTU data, and thus whether bacterial community profiles generated by 454 pyrosequencing could discriminate between the seven sample categories defined in this study. The Similarity Percentage (SIMPER) test (Clarke, 1993) was run on PAST software (Hammer et al., 2001) version 3.10 to identify which bacterial OTUs contributed the most to the (Bray-Curtis) dissimilarities observed among microhabitats.

## Results

### Dataset overview

In total, 113,260 raw 16S rRNA gene V4 sequence reads amplified from 24 TC-DNA samples were obtained. Of these, 82,400 passed quality filtering with AmpliconNoise (Quince et al., 2011). Further removal of post-filtering reads representing OTUs non-classifiable at the Domain level, or identified as chloroplasts or mitochondria, resulted in a total of 80,353 reads that constituted the analytical dataset. Altogether, these high-quality sequences were assigned to 1,953 OTUs at a 97% gene similarity cut-off, including 1,068 singleton OTUs classifiable at least at the Domain level (Table S1). These comprised 1.33% of the total number of analyzed reads.

### Bacterial richness and diversity

**Figure 2:**
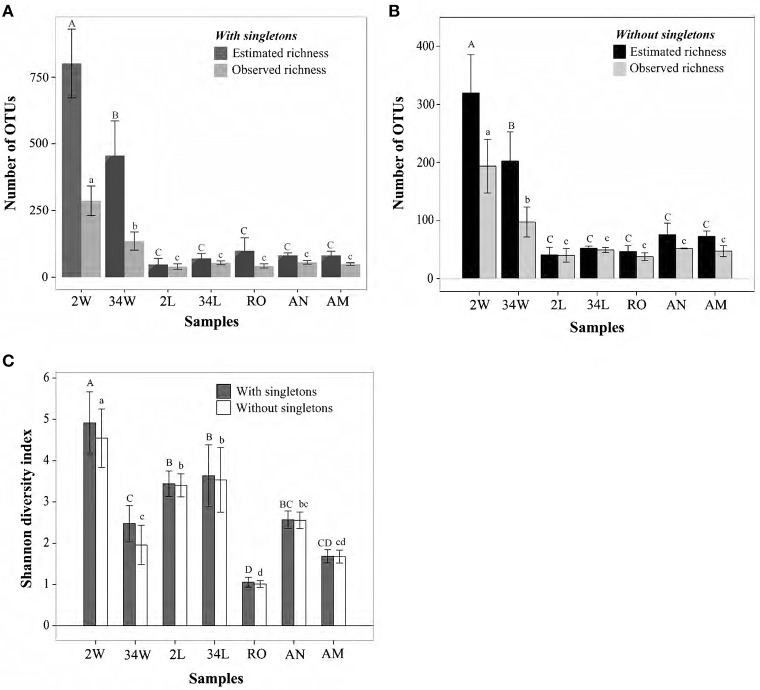
**Bacterial richness and diversity in gilthead seabream larviculture**. Observed and estimated (Chao1) richness measures when singleton OTUs are included **(A)** or excluded **(B)** are shown for size-normalized libraries (1,900 sequence reads per sample, the highest number of reads common to all samples), along with their respective Shannon diversity indices **(C)**. OTUs were determined at 97% 16S rRNA gene similarity, and values on bars represent means ± standard deviations of independent replicates within each sample category. Bars labeled with different letters represent statistically distinct sample categories in terms of richness and/or diversity values. In panels **(A,B)**, uppercase and lowercase letters define differences in estimated and observed richness, respectively, across sample categories. In panel **(C)**, they define differences in diversity indices across sample categories when singleton OTUs are included or ignored, respectively. Labeling of sample categories is as described in legend to Figure 1.

Differences in bacterial richness were statistically significant among the seven inspected sample categories (One Way ANOVA, *P* < 0.001, *DF* = 23, Figure 2A), with rearing-water samples displaying significantly higher values than host-associated samples (Figure 2A). Of note was a striking decrease in rearing-water bacterial richness during the trial, with averages (± standard deviation) of 286.5 ± 55.45 vs. 134.75 ± 34.05 OTUs detected per sample at 2 DAH and 34 DAH, respectively (Holm-Sidak *t* = 5.982, *P* < 0.001). In contrast, a subtle, non-significant (Holm-Sidak *t* = 2.371, *P* = 0.06) increase in richness was observed for fish larvae from 2 DAH (40 ± 10.14 OTUs) to 34 DAH (53.75 ± 7 OTUs) (Figure 2A). The live feed of fish larvae—rotifers, artemia nauplii and metanauplii—were similar in terms of bacterial richness (pairwise Holm-Sidak *t* < 1.830, *P* > 0.08, Figure 2A). Chao1 richness estimates retrieved for both 2 DAH and 34 DAH rearing-water were about 3-fold higher than the corresponding, observed bacterial richness values (Figure 2A). The difference between observed and estimated richness was not as pronounced, and often negligible, for the host-associated bacterial communities (Figure 2A). As expected due to their high richness values, bacterial communities from 2 and 34 DAH tank water were the most susceptible to the removal of singleton OTUs (Table 1, Figure 2B), averaging 193.5 ± 41.16 and 97.25 ± 25.90 OTUs per sample, respectively (Figure 2B). Reduction in richness values were not as pronounced in host-associated samples (Figure 2B) and, in comparative terms, both datasets with and without singletons revealed the same trends concerning shifts in bacterial richness across sample categories (Figures 2A,B). Shannon diversity indices—which consider not only the number of bacterial phylotypes (i.e., OTUs) but also their relative abundances in each sample—obtained for fish larvae (2 and 34 DAH) were, usually, significantly higher than those registered for the live feed (Figure 2C), suggesting greater equitability among community members in fish larvae than in the live feed. Further, at 34 DAH bacterial community diversity in the rearing-water was even lower than fish larvae diversity (Holm-Sidak *t* = 3.247, *P* = 0.005; Figure 2C) in spite of the much higher richness values registered for 34 DAH rearing-water in comparison with larvae (Figure 2A). Diversity estimates did not change significantly after removal of singleton OTUs (Figure 2C).

**Table 1:** **Number of bacterial sequences and OTUs detected per phylum across fish larviculture microhabitats**.

**Phylum**	**2W**		**34W**		**2L**		**34L**		**RO**		**AN**		**AM**	
	**seqs**	**OTUs**	**seqs**	**OTUs**	**seqs**	**OTUs**	**seqs**	**OTUs**	**seqs**	**OTUs**	**seqs**	**OTUs**	**seqs**	**OTUs**
*Acidobacteria*	34	19	6	5	0	0	0	0	0	0	0	0	0	0
*Actinobacteria*	146	26	15	8	6	2	14	6	4	3	0	0	4	4
*Bacteroidetes*	6,990	188	10,863	120	7	2	304	10	427	24	6,080	27	912	25
BHI80-139	0	0	2	2	0	0	0	0	0	0	0	0	0	0
*Chlorobi*	6	3	0	0	0	0	0	0	0	0	0	0	0	0
*Chloflexi*	10	6	9	7	0	0	0	0	2	2	0	0	0	0
*Cyanobacteria*	8	7	6	5	0	0	0	0	1	1	53	1	76	1
*Elusimicrobia*	0	0	1	1	0	0	0	0	0	0	0	0	0	0
*Firmicutes*	38	23	18	11	373	8	2,273	32	85	5	61	8	110	9
*Fusobacteria*	0	0	1	1	0	0	17	2	0	0	0	0	0	0
*Gemmatimonadetes*	7	4	2	2	0	0	0	0	0	0	0	0	0	0
GN02	51	22	2	2	1,005	4	0	0	72	3	303	2	0	0
*Lentisphaerae*	3	3	1	1	0	0	0	0	0	0	0	0	0	0
*Nitrospirae*	1	1	1	1	0	0	0	0	0	0	0	0	0	0
NKB19	1	1	0	0	0	0	0	0	0	0	0	0	0	0
OD1	28	21	6	4	22	1	0	0	0	0	0	0	0	0
OP11	34	23	2	2	0	0	0	0	0	0	0	0	0	0
OP3	39	26	7	6	0	0	0	0	0	0	0	0	0	0
PAUC34f	1	1	0	0	0	0	0	0	0	0	0	0	0	0
*Planctomycetes*	193	109	59	47	0	0	120	10	5	3	7	4	4	1
*Proteobacteria*	5,887	681	1,121	312	6,574	65	8,784	71	12,499	68	3,474	71	10,585	75
SAR406	4	3	1	1	0	0	0	0	0	0	0	0	0	0
SBR1093	4	2	2	1	0	0	6	1	0	0	0	0	0	0
*Spirochaetes*	8	6	1	1	0	0	0	0	0	0	0	0	0	0
SR1	1	1	0	0	0	0	0	0	0	0	0	0	0	0
*Tenericutes*	2	2	2	2	0	0	0	0	0	0	0	0	0	0
Thermi	4	2	0	0	0	0	0	0	0	0	0	0	0	0
TM6	70	37	20	15	0	0	5	2	0	0	0	0	0	0
TM7	22	10	1	1	0	0	0	0	0	0	0	0	0	0
*Verrucomicrobia*	64	30	244	12	0	0	0	0	4	1	0	0	0	0
WPS-2	4	2	0	0	0	0	0	0	0	0	0	0	0	0
WS3	3	2	3	3	0	0	0	0	0	0	0	0	0	0
ZB3	7	4	7	6	0	0	0	0	0	0	0	0	0	0
Unclassified	2	2	0	0	0	0	0	0	0	0	0	0	0	0
Total^a^	13,672	1,267/680	12,403	579/284	7,987	82/5	11,523	134/23	13,099	110/29	9,978	113/23	11,691	115/24

*Values correspond to quality-filtered OTUs and sequences across the full data set (non-normalized libraries, singleton OTUs included). 2 and 34 W, rearing-water sampled 2 and 34 days after hatching, respectively; 2 and 34 L, seabream larvae sampled 2 and 34days after hatching, respectively; RO, rotifers; AN, Artemia salina nauplii; AM, Artemia salina metanauplii; seqs, sequences*.

a *Shown are both the total number of OTUs / number of singleton OTUs detected in each sample category*.

### Bacterial community composition at the phylum and genus levels

**Figure 3:**
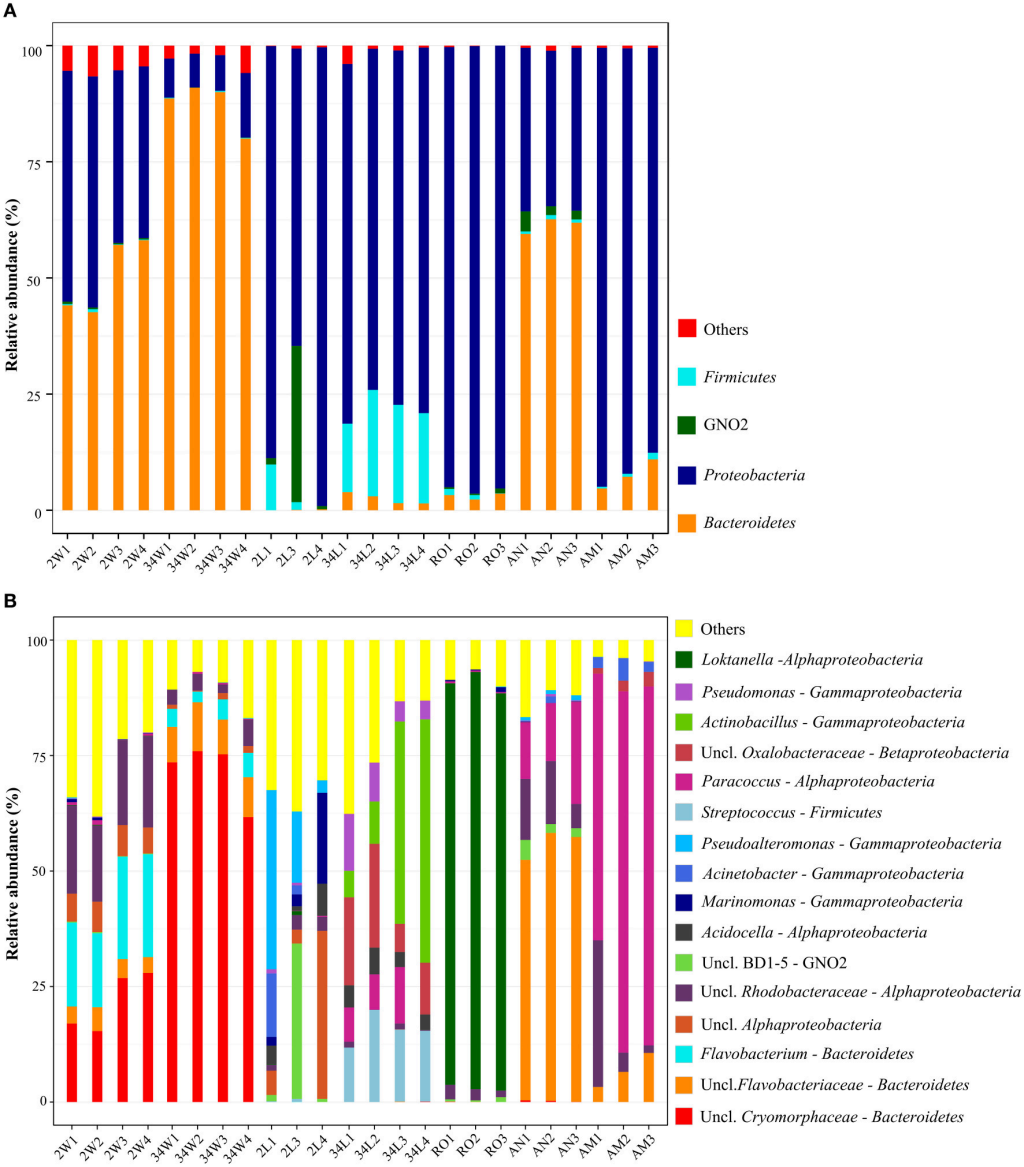
**Phylum-(A)** and genus-level **(B)** bacterial taxonomic composition in gilthead seabream larviculture. Results obtained for each replicate sample across all sample categories are shown, using size-normalized sequence libraries (1,900 reads per sample, singleton OTUs included). In both panels **(A,B)**, relative abundances are displayed only for taxa representing more than 1% of the total dataset reads. Taxa with abundances below 1% across the data are collectively labeled as “others.” Labeling of sample categories is as described in legend to Figure 1.

Three bacterial phyla, *Proteobacteria, Bacteroidetes* and *Firmicutes* (Table 1), accounted for more than 98.5% of all the retrieved quality-filtered sequence reads, with their relative abundances varying at a larger extent than would be expected by chance across the seven sample categories (Kruskal-Wallis, overall *P* < 0.03, *DF* = 6, Figure 3A. See Table S2, Supplementary Material, for details). *Proteobacteria* was clearly the dominant phylum in larvae- (2 and 34 DAH), rotifers-, and artemia metanauplii-associated bacterial communities, with average relative abundances of 83.81 ± 17.8%, 76.44 ± 2.25%, 95.46 ± 0.80%, and 91.07 ± 3.68%, respectively. Conversely, shared dominance between *Bacteroidetes* and *Proteobacteria* was observed in 2 DAH rearing-water (50.49 ± 8.25% and 43.38 ± 7.31%, respectively) and artemia nauplii (61.33 ± 1.65% and 34.57 ± 0.96%, respectively) samples (Figure 3A). A marked shift in community composition at the phylum level was registered for rearing-water during the trial as high *Bacteroidetes* dominance (87.39 ± 5.02%) was observed at 34 DAH. In fish larvae we detected a pronounced increase in *Firmicutes* abundance from 2 (3.82 ± 5.27%) to 34 DAH (19.55 ± 3.51%, Figure 3A). Finally, the until-recently candidate phylum GN02, now formally recognized as *Gracilibacteria* (Rinke et al., 2013), was apparently (see below) a characteristic phylum of early-stage fish larvae (2 DAH), occurring also–at moderate levels (2.70 ± 1.40%)—in artemia nauplii samples. In 2 DAH larvae, GN02 was represented mainly by one single OTU (OTU 2192, Table S1) and displayed high variability in relative abundance among samples (11.89 ± 18.83%, Figure 3A), showing therefore an inconsistent pattern of occurrence in this sample category. The proportions mentioned above remained largely unchanged when we explored relative abundances of phyla using the non-normalized dataset (Table 1).

The composition of the most dominant bacterial genera and unclassified families was markedly different among sample categories (Figure 3B), with relative abundances showing greater variations than could be predicted by chance (Kruskal-Wallis, overall *P* < 0.007, *DF* = 6. See Table S2 for details). A reduction in the number of genera was observed in rearing-water samples during the trial, coinciding with observations made at the OTU level (Figure 2). Indeed, most of the *Bacteroidetes* abundance in 34 DAH rearing-water could be attributed to a single OTU (OTU 268, Table S1) of the family *Cryomorphaceae*, which could not be classified at the genus level (Figure 3B). In contrast, 2 DAH rearing-water samples displayed a more balanced share between five genera in the *Bacteroidetes* and *Alphaproteobacteria* clades (Figure 3B), besides harboring several other, low abundance genera (Figure 3B, Table S1). Further, specific proteobacterial assemblages were identified in different larval developmental stages. Larvae sampled at 2 DAH were characterized by the genera *Marinomonas, Acinetobacter* and *Pseudoalteromonas* in the *Gammaproteobacteria* class, along with the genus *Acidocella* and an unclassified lineage (OTU 166, Table S1) in the *Alphaproteobacteria* class. Conversely, the genera *Pseudomonas, Actinobacillus* (*Gammaproteobacteria*), *Paracoccus* (*Alphaproteobacteria*) and *Streptococcus* (*Firmicutes*), in addition to a taxon (OTU 928, Table S1) of the *Oxalobacteraceae* family (*Betaproteobacteria*) tentatively affiliated with the genus *Massilia* (Table S3, Supplementary Material), prevailed in 34 DAH larvae (Figure 3B). Among live feed organisms, rotifers were dominated by a single OTU of the genus *Loktanella* (OTU 1801, Table S1), whereas artemia nauplii showed high abundances of unclassified *Flavobacteriaceae* (OTU 1902, Table S1), unclassified *Rhodobacteraceae* (OTUs 85 and 708, Table S1) and *Paracoccus* (*Alphaproteobacteria*, OTU 2374). The latter was the dominant genus in artemia metanauplii samples (Figure 3B). Usually, the most dominant genera / OTUs in the entire dataset (Table 2) displayed sharply variable abundance patterns, and therefore significantly contributed to differences in taxonomic composition among microhabitats (Table S3). Bacterial communities from early-stage larvae (2 DAH) displayed the highest level of variability in genus-level composition, especially regarding the relative abundances of *Pseudoalteromonas, Marinomonas*, unclassified *Alphaproteobacteria* and GN02 (Figure 3B).

**Table 2:** **Top ten most abundant bacterial OTUs^a^**.

**OTU ID**	**2W**	**34W**	**2L**	**34L**	**RO**	**AN**	**AM**	**Sum**	**Class**	**Order**	**Family**	**Genus**
268	1,635	5,421	0	2	0	0	0	7,058	*Flavobacteriia*	*Flavobacteriales*	*Cryomorphaceae*	
2,374	41	17	5	518	19	888	4,052	5,540	*Alphaproteobacteria*	*Rhodobacterales*	*Rhodobacteraceae*	*Paracoccus*
1,801	0	0	0	0	4,990	0	0	4,990	*Alphaproteobacteria*	*Rhodobacterales*	*Rhodobacteraceae*	*Loktanella*
1,902	3	1	0	0	3	3,167	287	3,461	*Flavobacteriia*	*Flavobacteriales*	*Flavobacteriaceae*	
1,461	0	0	0	2,116	0	0	0	2,116	*Gammaproteobacteria*	*Pasteurellales*	*Pasteurellaceae*	*Actinobacillus*
778	1,101	279	0	0	0	0	0	1,380	*Flavobacteriia*	*Flavobacteriales*	*Flavobacteriaceae*	*Flavobacterium*
928	0	0	0	1,118	0	0	127	1,245	*Betaproteobacteria*	*Burkholderiales*	*Oxalobacteraceae*	
1,019	1,070	130	0	0	0	0	0	1,200	*Alphaproteobacteria*	*Rhodobacterales*	*Rhodobacteraceae*	
1,296	5	4	1,080	0	3	51	1	1,144	*Gammaproteobacteria*	*Vibrionales*	*Pseudoalteromonadaceae*	*Pseudoalteromonas*
708	1	36	0	7	76	308	610	1,038	*Alphaproteobacteria*	*Rhodobacterales*	*Rhodobacteraceae*	

a *Top ten OTUs were defined using normalized libraries set at 1,900 sequences per sample to avoid deviations caused by higher or lower sequencing effort per sample category. Values correspond to the total number of sequence reads assigned to the most abundant OTUs when replicates of each sample category were pooled. Sample labels are as in legend to Table 1*.

### Ordination of bacterial OTUs

**Figure 4:**
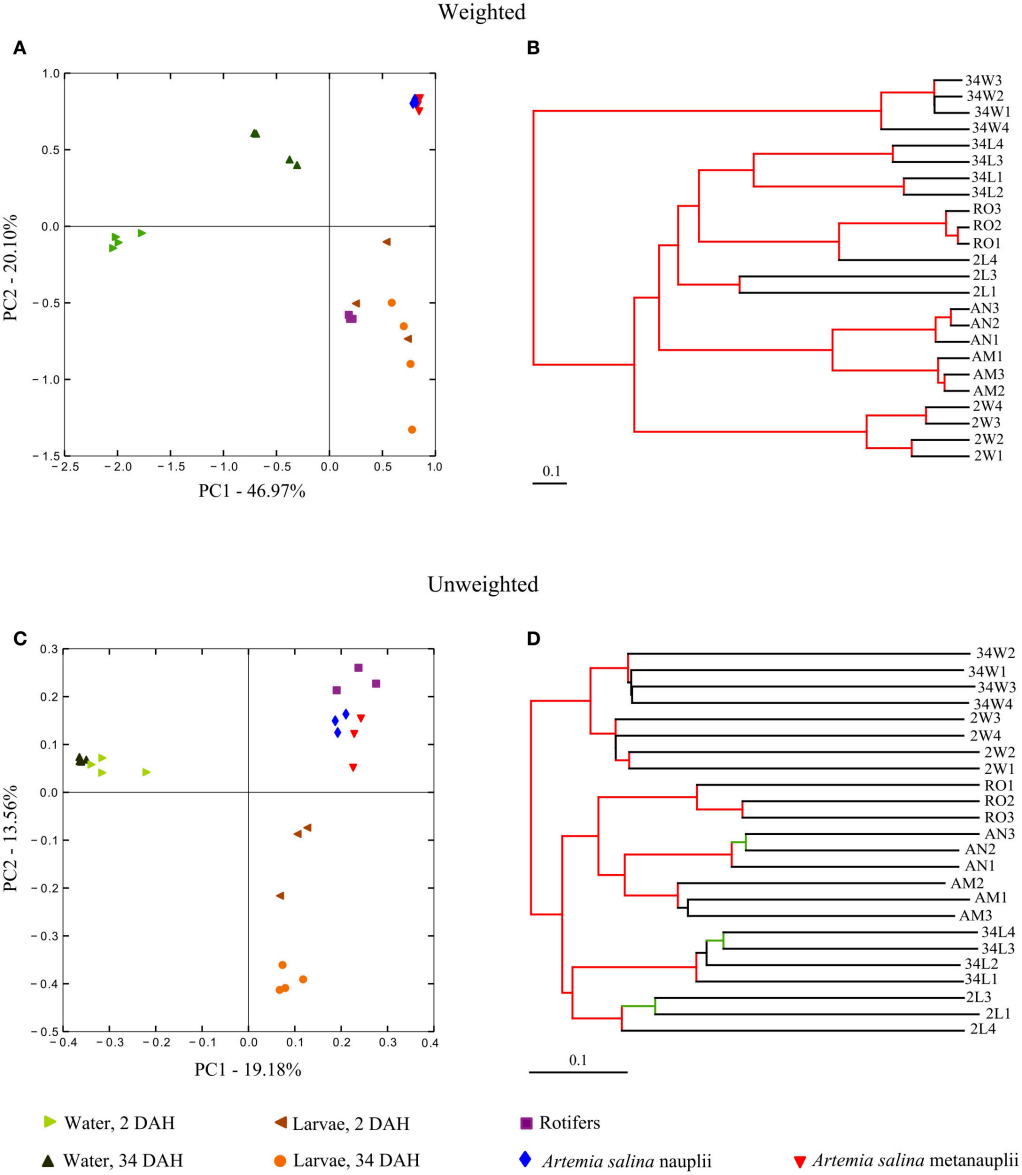
**Phylotype (OTU)-level ordination of bacterial communities in gilthead seabream larviculture**. Principal coordinates analyses (PCoA) were performed with weighted **(A)** and unweighted **(C)** Unifrac measures applied to size-normalized sequence libraries (1,900 reads per sample, singleton OTUs included). Corresponding cluster analyses performed on Unifrac measures using the UPGMA algorithm are displayed next to the PCoA plots **(B,D)** to reveal the discrete grouping of samples from the same similarity matrix. The robustness of the clusters was assessed by means of a jackknifed beta diversity permutation test and are revealed in the UPGMA dendrograms. Cluster nodes with bootstrap values above 75% are marked in red. Cluster nodes with bootstrap values between 50 and 75% are marked in green. Labeling of sample categories is as described in legend to Figure 1. See Figure S1 for analyses performed after exclusion of singleton OTUs from the dataset.

At the approximate “species” level of taxonomic resolution (OTUs), Principal Coordinates Analysis (PCoA) and Jackknifed UPGMA clustering performed on weighted (Figures 4A,B) and unweighted (Figures 4C,D) Unifrac measures were used to inspect the continuous (PCoA) vs. discrete (UPGMA) grouping of samples according to their degrees of OTU-community (dis)similarity. Altogether, these analyses revealed discrete grouping of replicates from each sample category into separate clusters with statistical support (Figures 4B,D), corroborating trends revealed by genus-level inspection of taxonomic composition (Figure 3B). The only exception to discrete clustering per microhabitat was one replicate from 2 DAH larvae (2L4), which first grouped with one cluster encompassing all rotifer replicates in the weighted cluster analysis (Figure 4B). Although grouping patterns were the same in both weighted and unweighted analysis, the extent of dissimilarity between the sample categories was consistently larger in the latter comparison. Trends revealed by community ordination remained unchanged when analyses were undertaken in the absence of singleton OTUs (Figure S1, Supplementary Material).

### Specific and shared OTUs across microhabitats

**Figure 5:**
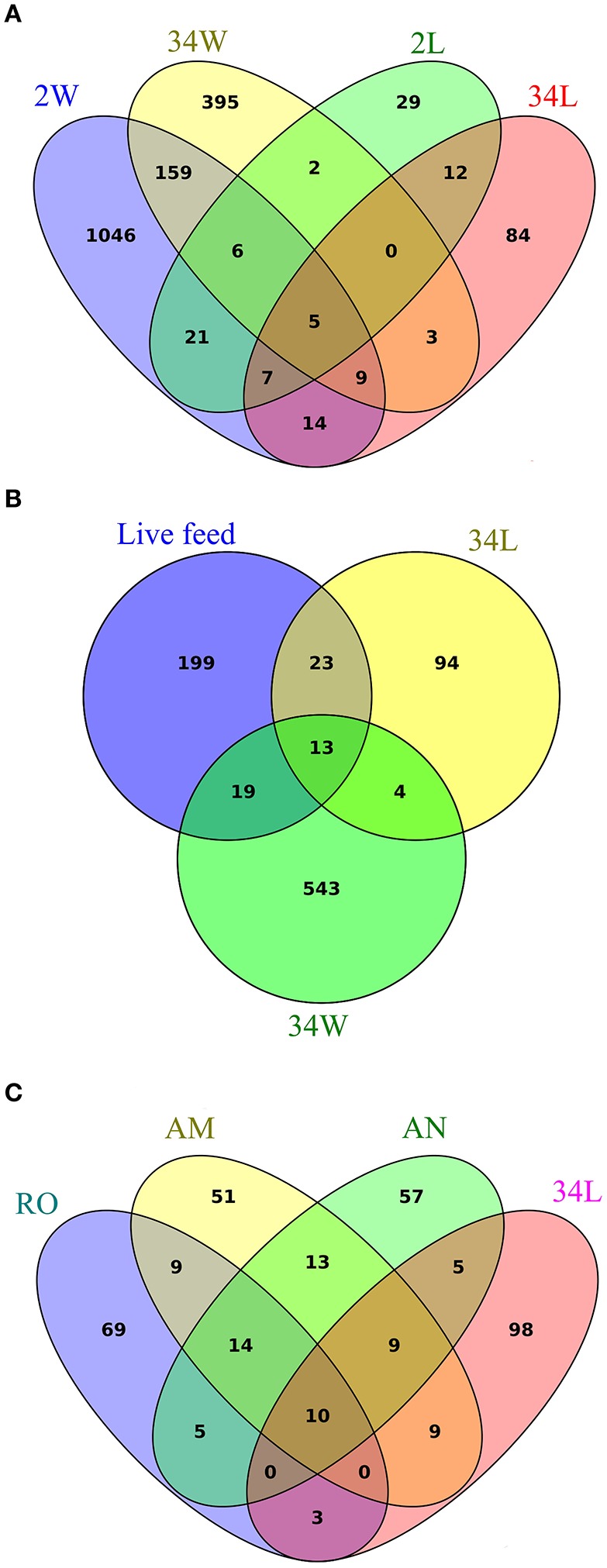
**Sharedness and specificity of bacterial phylotypes in gilthead seabream larviculture**. Venn diagrams were constructed exploring non-normalized libraries, considering all detected bacterial OTUs. Replicate samples were pooled to portray the total number of bacterial phylotypes recovered within each sample category. Diagram **(A)** enumerates OTUs common and exclusive to rearing-water (2 W, 34 W) and seabream larvae (2 L, 34 L) sampled 2 and 34 days after hatching, respectively. Diagram **(B)** displays the extent of OTU sharedness and exclusiveness between rearing-water (34 W) and seabream larvae (34 L) from 34 DAH, and the live feed used in the rearing trial (pooled samples of rotifers, *Artemia* nauplii and metanauplii). Diagram **(C)** further discriminates OTUs shared by and exclusive to rotifers (RO), *Artemia* sp. nauplii (AN), *Artemia* sp. metanauplii (AM) and seabream larvae sampled 34 days after hatching. Labeling of sample categories is as described in legend to Figure 1. See Figure S2 for analyses performed after exclusion of singleton OTUs from the dataset.

To determine how many and which OTUs were common or specific to each sample category, we explored the full (non-normalized) quality-filtered dataset using Venn diagrams where replicate samples per category were pooled (Figure 5). Quite surprisingly, only five bacterial OTUs were common to all 2 and 34 DAH rearing-water and fish larvae sample categories (Figure 5A). Larvae at 2 DAH hosted 29 specific OTUs within these four sample categories and shared 39 bacterial OTUs with its primary surrounding environment, that is, 2 DAH rearing-water (Figure 5A, Table S4, Supplementary Material). Only 17 OTUs were common to 34 DAH larvae and rearing-water from 696 OTUs detected in both sample types (Figure 5A, Table S5, Supplementary Material). Although several bacterial phylotypes shared by rearing-water and fish larvae corresponded to low or only moderately abundant OTUs across the data (Tables S4, S5), four of the 10 most abundant OTUs in the dataset (Table 2) were common to fish larvae and rearing-water samples. While OTUs 1296 (*Pseudoalteromonas*) and 2374 (*Paracoccus*) appeared to be enriched in 2 and 34 DAH fish larvae, respectively, presenting only very low numbers in the corresponding rearing-water samples, OTUs 268 (*Cryomorphaceae*) and 708 (*Rhodobacteraceae*) occurred in higher abundance in rearing-water, and thus appeared to be de-selected in the fish host (Table 2, Tables S4, S5). Further, the divergence between bacterial community structures in rearing-water from 2 and 34 DAH (see e.g., Figure 4) was also well illustrated, as a much larger pool of bacterial OTUs specific to, than shared by, both sample categories could be depicted (Figure 5A). The dichotomy between 2 and 34 DAH larvae-associated bacterial communities was also evident, as only 24 OTUs were common to both sample categories, while 58 and 110 OTUs from 2 and 34 DAH larvae, respectively, remained exclusive to each (Figure 5A).

To more specifically address the relative contribution of live feed and rearing-water as bacterial vectors for mature fish larvae, a Venn diagram was constructed using OTUs detected in the live feed (pooling of rotifers and artemia nauplii and metanauplii samples), 34 DAH rearing-water, and 34 DAH fish larvae as discrete OTU pools (Figure 5B). We further built one diagram in which the live feed was divided into rotifers, artemia nauplii and artemia metanauplii as single categories to determine their own unique contribution to shaping bacterial communities in 34 DAH larvae (Figure 5C). We found that a minority portion (36) of the OTUs documented in the live feed was also present in fish larvae approaching the juvenile stage (Figure 5B, Table S6, Supplementary Material), with only 10 such OTUs being simultaneously present in fish larvae and each of the live feed used in larval rearing (Figure 5C, Table S6). Nevertheless, we were able to single out several examples of strong selection of bacterial phylotypes in fish larvae from within this fraction of shared OTUs. This was certainly the case for OTUs 928 (unclassified *Oxalobacteraceae*), 705 and 1953 (*Pseudomonas*), 84 (unclassified *Enterobacteriaceae*), 807 (*Bradyrhizobium*), 1387 (*Sphingomonas*), and 1970 (*Acidocella*). All these OTUs displayed enhanced numbers in 34 DAH fish larvae but occurred at very low abundances in the live feed (Table S6).

Because OTUs represented by one read are by definition sample-specific, removal of singletons from the data led to a substantial reduction in the number of OTUs exclusive to each sample category, whereas the number of OTUs common to all sample categories remained unchanged (Figures S2A–C). However, the effect caused by singleton exclusion did not erode the picture of a larger pool of OTUs specific to than shared by sample categories (Figures S2A–C).

## Discussion

Bacterial communities in fish larviculture constitute a large reservoir of genetic and metabolic diversity that should not be undervalued in management practices. Indeed, in this study we detected 1,953 OTUs in seven sample categories—all of which characterized by highly distinct bacterial taxonomic profiles—in a gilthead seabream hatchery. About 90% of the singleton OTUs included in our analysis (Table S1), classified at least at the Domain level with the BLAST taxonomy assigner, could as well be classified as bacterial taxa using either the Greengenes or SILVA assignment methods (verified on December 06 2016). Encompassing 1,068 OTUs, these singletons represented a significant fraction of the bacterial richness captured in this survey, but a rather negligible proportion (1.3%) of the total number of analyzed reads. Indeed, we found that several sample categories, namely the live feed and 34 DAH rearing-water bacterial communities, were dominated by very few bacterial phylotypes (OTUs). In contrast, we verified that additional bacterial richness is likely to be revealed especially in rearing-water samples if further sequencing effort is applied, as suggested by the difference between observed and estimated richness values obtained for these consortia (Figures 2A,B). Because the methodology employed here enabled us to uncover highly diverse bacterial consortia particularly in fish larvae and 2 DAH rearing-water (and also in other marine symbiotic consortia–see e.g., Hardoim et al. (2014), the lower diversity/high dominance observed in the live feed and 34 DAH rearing-water is most likely indicative of a non-natural pattern of bacterial community structuring. This could result from strong selective pressures exerted on microbial populations in severely manipulated ecosystems. Rotifers and artemia used in fish larviculture are commonly maintained in captivity under long-term, steady conditions (e.g., kept in microalgal cultures at 15–18°C Ferreira, 2009). This is likely to reduce the diversity of their naturally occurring microbial consortia, eventually contributing to the prevalence of fewer microorganisms, in these hosts, that are able to cope with the processing and maintenance procedures. Our results contrast high bacterial richness measures obtained elsewhere for the live feed used in cod larval rearing (Bakke et al., 2015), likely reflecting the different sampling strategies employed in these studies, since we opted for collecting the live feed prior to their addition to the rearing tanks. The decrease in bacterial diversity observed in 34 DAH rearing-water could derive not only from the selection of populations performing well under controlled parameters but also from the presumably higher amounts of organic matter in larviculture tanks at late rearing stages. Accumulation of larval metabolic waste products via defecation, increase of dead biomass, be it from microalgae, food items or the larvae themselves, and the density of the microalgae daily applied to the rearing tanks may all contribute to higher loads of organic matter in fish farming (Vadstein et al., 2013). In spite of the high water renewal rates employed in our trial, all these increments influence the quality and quantity of the dissolved and particulate organic matter present in rearing tanks, thus most likely playing an important role in the selection of (fewer) microorganisms prevailing under the presumed, more eutrophic conditions at late larval rearing stages. What highlights the reduction in bacterial diversity from 2 DAH to 34 DAH rearing-water is an increase in abundance (from 21.8 to 71.6%, Figure 3, Table 2) of one single OTU affiliated with the *Cryomorphaceae* family (*Bacteroidetes*). Members of this family play a role in marine secondary production and require complex carbon sources for growth, being usually found in association with phytoplankton blooms and in environments rich in organic carbon (Zhou et al., 2013; Bowman, 2014). Although not verified experimentally, the increase in *Cryomorphaceae* in rearing-water during the trial may correlate with the cumulative introduction of microalgae to the tanks. Indeed, the total *Cryomorphaceae* abundance in 2 DAH rearing-water (*c*. 23%) can already be considered quite high, surpassing by 10-fold the amount of *Cryomorphaceae* found in the natural input water (i.e., water from the Ria Formosa lagoon, Olhão; Costa et al., unpublished data).

Besides the rather sharp increase in abundance of *Cryomorphaceae* in the rearing-water during the experiment, several other OTUs could be identified as distinguishing bacterial taxa among microhabitats. The genera *Loktanella* (dominant in rotifers) and *Paracoccus* (dominant in artemia metanauplii) are bacterial groups in the *Rhodobacteraceae* family (*Alphaproteobacteria*) with potential probiotic activity (Hjelm et al., 2004; Makridis et al., 2005; Yan et al., 2014). These taxa were abundant in the live feed but were very scarce in rearing-water and fish larvae. Higher *Loktanella* spp. proportions were found on fronds of the macroalga *Ulva australis* than in seawater (Burke et al., 2011), and they ranked as the prevailing culturable bacteria associated with laboratory strains of microalgae (Schwenk et al., 2014). It is possible, therefore, that *Loktanella* spp. accumulate within rotifers after digestion of microalgae besides water filtering with both processes playing a role in maintaining this consistent association in fish larviculture. Besides its pronounced dominance in artemia metanauplii, the genus *Paracoccus* was quite abundant in artemia nauplii, but only moderately abundant in 34 DAH larvae. The sole, but highly abundant, *Paracoccus* phylotype (OTU 2374) found in artemia metanauplii displays closest 16S rRNA gene relatedness with *P. zeaxanthinifaciens* (Table S3), a bacterium isolated from seaweed found to produce the yellow carotenoid zeaxanthin, a compound applied in poultry pigmentation and in the prevention of age-related degeneration in humans (Berry et al., 2003). Its low abundance in rearing-water (both at 2 and 34 DAH) suggests that this strain may accumulate into live feed tissue (especially *Artemia* spp.) through filtering activity and then pass onto fish larvae through feeding. However, it tends not to be present in such high densities in the fish host as observed in the live feed, but rather to be a regular, constituent member of a more diversified fish bacterial consortium. In the specific context of our gilthead seabream larval rearing, both *Loktanella* and *Paracoccus* are, apparently, less likely to possess crucial relevance to larval physiology and metabolism since their occurrence in association with the host was not favored.

Owing to the high-throughput nature of our analysis, we were able to unmask several bacterial populations (OTUs) whose distribution across the studied microhabitats exemplifies a mode of bacterial acquisition characterized by sharp enrichment, within host tissues, of otherwise extremely low abundant populations in the live feed or in the environmental surroundings. For instance, two *Pseudomonas* OTUs (705 and 1953) contribute to the quite high abundance of this genus in mature fish larvae. They display close phylogenetic relationship with *P. fragi* (Miller et al., 1973; Cormier et al., 1991) and *P. lini* (Delorme et al., 2002), respectively, and extremely low abundances in live feed samples (Table S1). Although the life-strategy of *P. fragi* and relatives is suggestive of typical opportunistic behavior with pathogenic potential, there is currently no evidence for the participation of either *P. fragi* and *P. lini* as aethiological agents of disease in fish. Likewise, OTU 928 (family *Oxalobacteraceae*, order *Burkholderiales*) (Baldani et al., 2014), was an abundant phylotype in 34 DAH larvae also present in all artemia metanaupli replicates, albeit at only negligible densities. The *Oxalobacteraceae* family is metabolically diverse and includes strict anaerobes, aerobes, and nitrogen-fixing organisms. Phylogenetic inference suggests that OTU 928 is a member of the genus *Massilia* (Table S3), a relatively widespread taxon registered in soils, soil crusts, air and humans (La Scola et al., 1998; Ferrari et al., 2005; Gundlapally and Garcia-Pichel, 2006; Kämpfer et al., 2011). Our results indicate a classical enrichment of this phylotype in fish larvae through live feed ingestion. Its prevalence at the later larval stage is well justified by its presence in *Artemia metanauplii* samples only, even if at low abundances. These data fit well previous observations on the occurrence of *Oxalobactaraceae* in the intestinal tract of sea bass juveniles (Carda-Dieguez et al., 2014). A further dominant taxon in 34 DAH larvae, which was however absent in all other microhabitats, the genus *Actinobacillus* (*Gammaproteobacteria, Pasteurellaceae*) contains species recognized as parasites or pathogens of mammals, birds and reptiles (Slots and Ting, 1999; Kuhnert and Christensen, 2008; Macinnes et al., 2012). *Actinobacillus* spp. have already been documented in aquaculture ponds (Ampofo and Clerk, 2003), and there is no current evidence of their role as fish pathogens. Similarly, we observed two OTUs (1128 and 2143) that primarily contribute to the abundance of the genus *Streptococcus* (*Firmicutes*) in 34 DAH larvae, but were detected neither in water nor in live feed samples. Several *Streptococcus* spp. are known to cause disease in fish, and *S. iniae* is a leading pathogen in aquaculture worldwide (Baiano and Barnes, 2009). Phylogenetic inference indicates that our OTUs are more closely related with the human pathogenic species *S. dysgalactiae* and *S. infantis*/*mitis* (Table S3). Particularly, *S. dysgalactiae* has been recently recognized as an emerging pathogen infecting a wide variety of fish species (Abdelsalam et al., 2013), causing *e.g*., necrosis in the caudal peduncles and high mortality rates in cultured amberjack (*Seriola dumerili*) and yellowtail (*Seriola quinqueradiata*) (Nomoto et al., 2004). Because we did not detect *Streptococcus* and *Actinobacillus*-related sequences in live feed and tank water, the actual source(s) of these phylotypes to the fish larvae could not be verified. However, this is likely to be overcome in future studies employing greater sequencing output. For *Streptococcus* spp. particularly, their presence in 2 DAH larvae suggests that they are early fish colonizers with the ability to persist and eventually increase in abundance as the host develops. This could either result from high competitive capacity within the emerging fish (gut) microbiome or cumulative host colonization from low abundant, environmental populations, or through both mechanisms simultaneously.

Our data strengthen previous observations concerning the disparity between bacterial community profiles from fish larvae and their corresponding live feed (Bakke et al., 2013, 2015). However, we posit that the latter are actual participants in shaping the fish (larvae) microbiome and might bear importance as latent vectors of bacterial associates of fish. Here, we reveal several bacterial phylotypes that occurred at negligible abundances in the live feed, but were specifically selected for in fish larvae. In fact, such a pattern of bacterial enrichment—of either mutualists, pathogens or commensals—in, or on the surface of, eukaryotes is common across several host-microbe interactions in aquatic ecosystems (Webster et al., 2010; Simister et al., 2012; Costa et al., 2013; Hardoim et al., 2014; Cúcio et al., 2016). It may be driven by diverse mechanisms, such as host filtering/drinking activity, parental symbiont transmission and host-derived chemical cues, besides the high doubling rates of opportunistic bacteria during favorable conditions. Altogether, all seven sample categories represented microbial communities that significantly differ in structure (Figure 4 and Figure S1), even if only presence/absence OTU data are considered (Figure 4D and Figure S1D). The extent of between-replicate variability in community distance measures, within any given sample category, was much reduced when OTU relative abundances were considered in both datasets with and without singletons (Figure 4B and Figure S1B), highlighting the importance of taxon abundance ranks in determining consistency in community assembly patterns.

We here describe the autochthonous bacterial consortium of early-stage gilthead seabream larvae as a quite diverse (Figure 2C), readily detectable community of prevalently alpha—and gammaproteobacterial lineages (Figure 3B) emerging prior to host's mouth opening and complex tissues' development. Therefore, they likely represent, to some extent, the assemblage of pioneering bacterial settlers on eggs. This assemblage is primarily formed by typical free-living, commensalistic or symbiotic marine bacteria (e.g., *Marinomonas, Acidocella, Pseudoalteromonas, Rhodobacteraceae*). Of note here is the high abundance of OTU 1296, which presented 100% 16S rRNA gene similarity with multiple species of the genus *Pseudoalteromonas* (e.g., *P. porphyrae, P.‘atlantica, P. undina, P. espejiana*, Table S3). *Pseudoalteromonas* spp. perform well as early colonizers of marine surfaces, eventually dictating bacterial succession in such substrates through the profuse biosynthesis of extracellular polysaccharides and enzymes, as is the case of widespread *P. atlantica* (Corpe, 1973; Holmström and Kjelleberg, 1999). Several *Pseudoalteromonas* strains, including representatives of *P. undina* and *P. espejiana*, were found to present no virulence toward gilthead sea-bream juveniles (Pujalte et al., 2007), and therefore we suggest that the interaction of these species with sea-bream larvae is rather of a commensalistic or mutualistic nature. The fish larvae-associated community shifts markedly in structure at 34 DAH, whereby genera such as *Pseudomonas, Actinobacillus, Streptococcus, Massilia*, and *Paracoccus* prevailed. It is likely that the above-mentioned changes are, to a considerable extent, driven by the higher degree of tissue compartmentalization, and thus distinct niche availability, in fully developed fish larvae. This could also partially explain the higher bacterial diversity found in 34 DAH than in 2 DAH larvae. Further, changes in the quality and quantity of available organic carbon that take place during larval rearing (Vadstein et al., 2013) certainly influence the dynamics and propagation of microorganisms in the system, possibly playing a role in the differential enrichment of bacterial phylotypes at early and late larval developmental stages observed here. Bearing in mind the limitations of 16S rRNA gene approaches in delivering accurate species-level identification, and therefore serving as proxies for pathogenicity among the bacteria (Martins et al., 2013), we here offer a cautious interpretation of potential symbiotic vs. pathogenic behavior derived from taxonomy data. Future cultivation-independent, functional studies of the fish larval microbiota, enabled e.g., via shotgun DNA sequencing, will be fundamental to more adequately address the relative proportions of mutualistic vs. pathogenic traits of bacterial associates at early vs. late larval rearing stages, advancing our current knowledge in this regard besides 16S rRNA gene-centered bacterial taxonomic profiling. Nevertheless, our methodological approach delivered a sound diagnosis of the status of the larva-associated bacterial communities and shifts thereof. Based on our results, we envision the fish host intermittently subjected to a succession of bacterial cohorts that shift in structure—composition, diversity, abundance—during larval rearing, substantially contributing to a differential recruitment of bacterial associates by fish larvae as the host develops.

Finally, the structure of bacterial communities populating fish hatcheries is likely to shift considerably in a case-by-case manner. Certainly, manifold factors, such as intrinsic features of the larviculture system itself, the quality and the indigenous microbiota of the water supply, the reared species, the chosen diet and environmental parameters, among others, are all supposed to play a role in shaping the larviculture microbial consortia. For instance, the assemblages of dominant bacterial genera reported here for gilthead seabream differ from those reported recently for cod larvae (Bakke et al., 2015), highlighting the relevance of the host species, among other factors, in shaping its symbiotic consortium. Therefore, continued research effort is needed for a broader understanding of the dynamics of these microbial communities across several model fish species and rearing conditions, if we are to effectively manipulate these assemblages for improved land-based fish larviculture. In this study, we diagnosed an intriguing pattern of host-driven enrichment and de-selection of bacterial phlotypes in both 2 and 34 DAH gilthead seabream larvae, highlighting the relative contribution of the environment (rearing-water) and the live feed as sources of bacteria, and of selective pressures, in shaping the microbiome of early-stage fish larvae. Determining the mutualistic or eventual pathogenic nature of these bacterial associates will lead to a much improved understanding of the relevance and dynamics of the fish larvae microbiome. In spite of the comprehensive approach employed in this study, future surveys approaching microbial diversity associated with other important components (“microhabitats”) of the system, such as the microalgae commonly used in the application of the “green water technique” can further enhance our knowledge of the phylogenetic breadth of the microbial consortia that are relevant to fish larval rearing.

## Ethics statement

This study was exempt from ethical approval procedures according to the current Portuguese legislation. This study did not occur within privately owned or protected areas. This study did not involve endangered or protected species.

## Author contributions

LR, LM, and RC designed the study; GC, SC, FS, LR, and RC performed the experiments; CC, LR, LM, and RC provided reagents and materials; GC, CC, and RC analyzed the data; GC and RC wrote the main manuscript text and prepared figures. All authors reviewed the manuscript.

## Funding

This work was supported by the Portuguese Foundation for Science and Technology through the research grants PTDC/MAR/112792/2009, UID/Multi/04326/2013 and UID/BIO/04565/2013. Further support was provided to the Institute for Bioengineering and Biosciences by “Programa Operacional Regional de Lisboa 2020” (Project N. 007317).

### Conflict of interest statement

The authors declare that the research was conducted in the absence of any commercial or financial relationships that could be construed as a potential conflict of interest.

## Supplementary material

Appendix S1 **Methodological details and extended discussion**.

Figure S1 **Phylotype (OTU)-level ordination of bacterial communities in gilthead seabream larviculture**. Analyses were performed after exclusion of singleton OTUs from the quality-filtered dataset. Details are as in legend to Figure 4.

Figure S2 **Sharedness and specificity of bacterial phylotypes in gilthead seabream larviculture**. Analyses were performed after exclusion of singleton OTUs from the quality-filtered dataset. Details are as in legend to Figure 5.

Table S1 **Distribution, abundance and taxonomic assignment of bacterial OTUs**.

Table S2 **Non-parametric statistics for relative abundance distributions of dominant taxa across sample categories**.

Table S3 **Top 25 most differentiating OTUs**.

Table S4 **Bacterial OTUs common to water and fish larvae (2 DAH)**.

Table S5 **Bacterial OTUs common to water and fish larvae (34 DAH)**.

Table S6 **Bacterial OTUs common to live feed and fish larvae (34 DAH)**.
