# Supplementary material for: Molecular Taxonomic Profiling of Bacterial Communities in a Gilthead Seabream (Sparus aurata) Hatchery
Source: Front Microbiol. 2017 Feb 14;8:204. doi: 10.3389/fmicb.2017.00204 (PMC5306143; doi:10.3389/fmicb.2017.00204)
Supplement: Appendix S1 — Methodological details and extended discussion. [file Presentation1.PDF]

Appendix S1 │Methodological details and extended discussion.

454 pyrosequencing of PCR amplicons

Electrophoresis of the PCR products was undertaken on a 1% (w/v) agarose gel and the 248 bp amplified fragments were purified using AMPure XP beads (Agencourt, Beckman Coulter, USA) according to the manufacturer's instructions. The amplicons were quantified with the PicoGreen dsDNA quantitation kit (Invitrogen, Life Technologies, Carlsbad, California, USA), pooled at equimolar concentrations and sequenced in the A direction with GS 454 FLX Titanium chemistry, following the manufacturer's instructions (Roche, 454 Life Sciences, Branford, CT, USA), at Genoinseq (Cantanhede, Portugal). The raw pyrosequencing reads were assigned to the appropriate samples based on the respective barcode (Table AppS1.1). Then, reads were quality filtered to minimize the effects of random sequencing errors, by elimination of sequence reads with <100 bp and sequences that contained more than two undetermined nucleotides (N).

Sample units and processing for bacterial community profiling

As fish microbiome research gains momentum (Llewellyn *et al.* 2014), standardization of methodological procedures may be desirable to enable broader data interpretation in an ecological context, taking into account diverse aquaculture settings and model host species. In this study, we used gilthead seabream larval pools as a sample unit. This way, we could more adequately compare early- and late-stage fish larvae, standardizing sample size by wet weight (0.2 g larval pools). However, approaching microbial communities in individual larvae is certainly advantageous for several reasons (Bakke *et al.* 2013), and this truly valid sampling strategy needs to be considered depending on experimental design. Preliminary experiments conducted in our laboratory demonstrated that DNA yields obtained from individual gilthead seabream larvae sampled two days after hatching (2 DAH) were very limiting (< 0.2 ng / µL) for downstream PCR-amplification and sequencing analysis. Furthermore, we observed that the 0.2 – 0.25 g wet weight range was suitable for the processing and DNA extraction from all other host-associated samples considered in the study (rotifers, artemia and 34 DAH larvae). Therefore, by using 0.2 g wet weight as starting material for DNA extraction, we delivered a sound comparison between all host-associated bacterial consortia through a robust and standardized methodology. Whereas the pooling of fish larvae does not permit inspection of microbiome dynamics at the individual level, it may be advantageous by enabling solid

verification of consistent and relevant trends regarding microbial community composition and diversity. It also enables appreciation of host-associated microbiome dynamics in a tractable fashion when dealing with small-sized animals. Especially when several experimental treatments need to be approached, tackling ecological problems at the (larval) individual level may render unfeasible (financially, experimentally and logistically) if we are to derive new hypotheses and diagnose major trends using community ecology data. It is important to emphasize that true biological replicates (in our case, the usage of four independent rearing-tanks) are strictly needed to support sound assessment of microbial community structures in fish larvae, live feed and the rearing-water (Bakke *et al.* 2013), regardless of whether sample units are animal individuals or pools. For example, in our study the variability in relative abundance of some bacterial taxa detected in 2 DAH larvae (*e.g.* GN02, *Pseudoalteromonas*, *Marinomonas* - Fig S1, Supporting information) suggests that stochastic events may be relevant for bacterial community assembly in early-stage fish larvae. This trend could only be determined because of the independent replication strategy employed in this study, whereas it is likely that the extent of variation observed would be greater if individual larvae had been investigated within and between tanks.

Several microbiological studies of fish larvae or tissue (*e.g.*, gut) use disinfecting agents such as benzalconiumchloride as a means of surface-sterilizing samples prior to processing. This strategy is useful especially when cultivation of microbes from internal tissues (*e.g.* gut of fish larvae) or gut contents is desired. In this study, we opted instead for washing fish larvae gently with artificial sterile seawater to remove loosely attached bacteria from the samples. As a primary metagenomics-based, cultivation-independent endeavour, the detection of DNA from cells damaged or killed by disinfection procedures would have been possible anyway if the material remained attached to larval surfaces. Furthermore, it is likely that the microbial consortium associated with the surface of 2 DAH gilthead seabream larvae is of utmost relevance for the establishment of the mature fish microbiome and the development of the host´s immune system. Indeed, at this stage the larvae still did not open their mouth, and gut development is very incipient. Therefore, we decided for a sample processing strategy conducive to the detection of both surface-associated and endosymbiotic bacteria in 2 and 34 DAH fish larvae.

Distribution of *Vibrionaceae* spp. across microhabitats

Usually regarded as some of the most relevant agents of disease in intensive fish rearing, members of the family *Vibrionaceae* such as *Vibrio* and *Photobacterium* spp. presented rather negligible to low abundances

in comparison with the most abundant taxa detected in this study. In fact, only one *Vibrionaceae* OTU (OTU 320, Table S1) displayed a pattern of occurrence across microhabitats that resembled that of other potentially pathogenic taxa such as *Pseudomonas*, *Oxalobacteraceae*, *Actinobacillus* and *Streptococcus* spp.. However, enrichment of OTU 320 was observed exclusively for 2 DAH larvae, and was much less pronounced than larval enrichment of the taxa highlighted above. In spite of their solid documentation as fish pathogens (Bergh 2000; Martins *et al.* 2015; Olafsen 2001; Vadstein *et al.* 2013), *Vibrio* and *Photobacterium* species are particularly amenable to domestication in the laboratory, and this may in part favour their widely accepted perception as the most prolific causes of bacterial infection in land-based fish rearing. Comprehensive, cultivation-independent studies of the fish microbiome will therefore be instrumental for a more balanced picture, in the near future, of the diversity of potentially pathogenic bacteria across several fish hatcheries.

Towards the analysis of the "total microbiome" in fish larviculture

In spite of the comprehensive approach employed in this study, future surveys shedding light on other relevant microbial groups such as archaea and fungi will certainly add further layers of complexity to, and increase our perception of, the "total" microbiome found in gilthead seabream larviculture and aquaculture settings as a whole. Importantly, additional bacterial diversity is likely to be captured, especially in rearing-water samples, if further sequencing effort is applied to investigate these consortia. Likewise, approaching microbial diversity associated with further, important components ("microhabitats") of the system, such as the microalgae commonly used in the application of the "green water technique" can further enhance our perspective of the phylogenetic breadth circumscribed within the microbial consortia that are relevant to fish larval rearing.

Table AppS1.1 8-mer barcodes used in this study

**Sample**	**Microhabitat**	**Sequencing tag**
2W1	Rearing-water, 2 DAH	AGAGATGC
2W2	Rearing-water, 2 DAH	AGAGCAGC
2W3	Rearing-water, 2 DAH	AGAGCATG
2W4	Rearing-water, 2 DAH	AGATCATC
34W1	Rearing-water, 34 DAH	AGATGCTC
34W2	Rearing-water, 34 DAH	AGCAGAGC
34W3	Rearing-water, 34 DAH	AGCAGATG
34W4	Rearing-water, 34 DAH	AGCAGCAG
2L1	Fish larvae, 2 DAH	AGATCTGC
2L3	Fish larvae, 2 DAH	AGATGATG
2L4	Fish larvae, 2 DAH	AGATGCAG
34L1	Fish larvae, 34 DAH	AGCTCATG
34L2	Fish larvae, 34 DAH	AGCTGATC
34L3	Fish larvae, 34 DAH	AGCTGCTG
34L4	Fish larvae, 34 DAH	ATCAGATC
RO1	Rotifers	GTCTATCA
RO2	Rotifers	TCATCTCT
RO3	Rotifers	TCTCGATC
AN1	Artemia nauplii	TGTATCTC
AN2	Artemia nauplii	AGTGAGAG
AN3	Artemia nauplii	GAGTCAGA
AM1	Artemia metanauplii	ATCGTACG
AM2	Artemia metanauplii	ACACTATG

AM3	Artemia metanauplii	TGCGTGCT

References

Bakke I, Skjermo J, Vo TA, Vadstein O (2013) Live feed is not a major determinant of the microbiota associated with cod larvae (Gadus morhua). *Environmental Microbiology Reports*, **5**, 537-548.

Bergh Ø (2000) Bacterial pathogens associated with early life stages of marine fish. *Microbial Biosystems: New Frontiers*, 221-228. Proceedings of the 8th International Symposium on Microbial Ecology. Bell CR, Brylinsky M, Johnson-Green P (ed). Atlantic Canada Society for Microbial Ecology, Halifax, Canada.

Llewellyn MS, Boutin S, Hoseinifar SH, Derome N (2014) Teleost microbiomes: the state of the art in their characterization, manipulation and importance in aquaculture and fisheries. *Frontiers in Microbiology*, **5**, 207.

Martins P, Navarro RV, Coelho FJ, Gomes NC (2015) Development of a molecular methodology for fast detection of Photobacterium damselae subspecies in water samples. *Aquaculture*, **435**, 137-142.

Olafsen JA (2001) Interactions between fish larvae and bacteria in marine aquaculture. *Aquaculture* **200**, 223-247.

Vadstein O, Bergh O, Gatesoupe F-J, *et al.* (2013) Microbiology and immunology of fish larvae. *Reviews in Aquaculture*, **5**, S1-S25.
