# Supplementary figures and images for: Molecular Taxonomic Profiling of Bacterial Communities in a Gilthead Seabream (Sparus aurata) Hatchery
Source: Front Microbiol. 2017 Feb 14;8:204. doi: 10.3389/fmicb.2017.00204 (PMC5306143; doi:10.3389/fmicb.2017.00204)

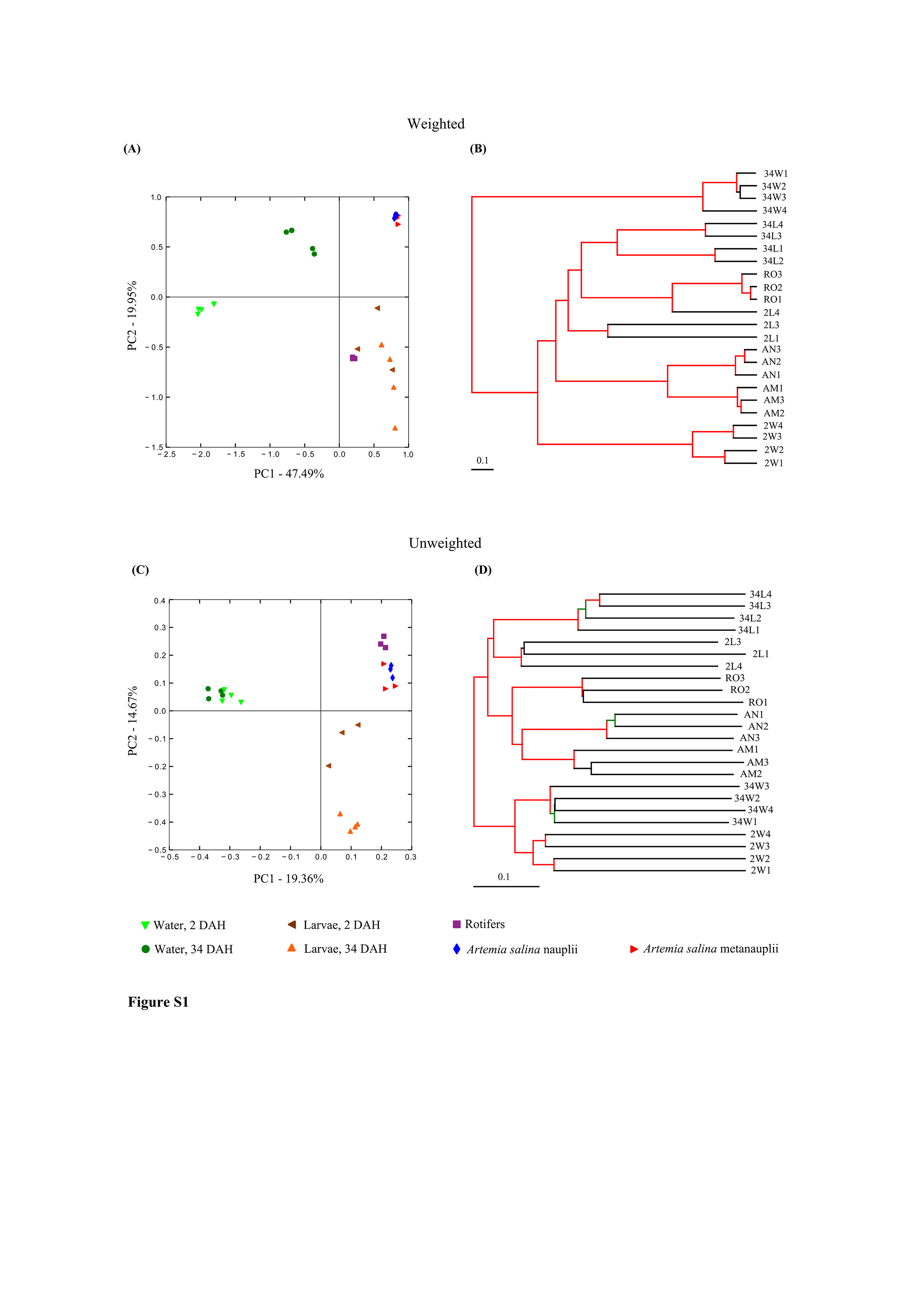

Supplement: Figure S1 — Phylotype (OTU)-level ordination of bacterial communities in gilthead seabream larviculture. Analyses were performed after exclusion of singleton OTUs from the quality-filtered dataset. Details are as in legend to Figure 4. [file Image1.tif]
