# Supplementary material for: Molecular Taxonomic Profiling of Bacterial Communities in a Gilthead Seabream (Sparus aurata) Hatchery
Source: Front Microbiol. 2017 Feb 14;8:204. doi: 10.3389/fmicb.2017.00204 (PMC5306143; doi:10.3389/fmicb.2017.00204)
Supplement: Table S4 — Bacterial OTUs common to water and fish larvae (2 DAH). [file Table4.DOCX]

**Table S4│Bacterial OTUs common to water and fish larvae (2 DAH).**

| **OTU ID** | **2W**  **(n=13672^1^)** | **2L**  **(n=7987^1^)** | **Phylum** | **Class** | **Order** | **Family** | **Genus** |
| --- | --- | --- | --- | --- | --- | --- | --- |
| 82 | 1 | 2 | *Proteobacteria* | *Gammaproteobacteria* | *Oceanospirillales* | *Oceanospirillaceae* | *Oceanospirillum* |
| 85 | 267 | 9 | *Proteobacteria* | *Alphaproteobacteria* | *Rhodobacterales* | *Rhodobacteraceae* |  |
| 124 | 2 | 1 | *Proteobacteria* | *Gammaproteobacteria* | *Alteromonadales* |  |  |
| 166 | 3 | 848 | *Proteobacteria* | *Alphaproteobacteria* |  |  |  |
| 183 | 1 | 33 | *Proteobacteria* | *Gammaproteobacteria* | *Alteromonadales* | *Alteromonadaceae* | BD2-13 |
| 320 | 12 | 109 | *Proteobacteria* | *Gammaproteobacteria* | *Vibrionales* | *Vibrionaceae* |  |
| 423 | 2 | 5 | *Bacteroidetes* | *Flavobacteriia* | *Flavobacteriales* | *Cryomorphaceae* | *Fluviicola* |
| 437 | 3 | 147 | *Proteobacteria* | *Gammaproteobacteria* | *Alteromonadales* | *Colwelliaceae* |  |
| 447 | 0 | 13 | GN02 | BD1-5 |  |  |  |
| 568 | 1 | 185 | *Proteobacteria* | *Alphaproteobacteria* | *Rhizobiales* | *Methylobacteriaceae* | *Methylobacterium* |
| 627 | 0 | 7 | *Proteobacteria* | *Alphaproteobacteria* | *Rhizobiales* | *Rhizobiaceae* | *Agrobacterium* |
| 747 | 30 | 119 | *Proteobacteria* | *Alphaproteobacteria* | *Rhodobacterales* | *Rhodobacteraceae* | *Phaeobacter* |
| 770 | 0 | 8 | *Proteobacteria* | *Alphaproteobacteria* | BD7-3 |  |  |
| 779 | 2 | 291 | *Proteobacteria* | *Gammaproteobacteria* | *Pseudomonadales* | *Moraxellaceae* | *Acinetobacter* |
| 807 | 3 | 108 | *Proteobacteria* | *Alphaproteobacteria* | *Rhizobiales* | *Bradyrhizobiaceae* | *Bradyrhizobium* |
| 820 | 0 | 79 | *Proteobacteria* | *Alphaproteobacteria* | *Rhodobacterales* | *Rhodobacteraceae* |  |
| 923 | 1 | 14 | *Firmicutes* | *Bacilli* | *Bacillales* | *Alicyclobacillaceae* | *Alicyclobacillus* |
| 1112 | 7 | 18 | *Proteobacteria* | *Alphaproteobacteria* | *Rhodobacterales* | *Rhodobacteraceae* | *Loktanella* |
| 1179 | 4 | 15 | *Proteobacteria* | *Gammaproteobacteria* | *Alteromonadales* | *Alteromonadaceae* | *Alteromonas* |
| 1255 | 56 | 243 | *Proteobacteria* | *Gammaproteobacteria* | *Oceanospirillales* | *Oceanospirillaceae* | *Oleispira* |
| 1269 | 1 | 0 | *Proteobacteria* | *Gammaproteobacteria* | *Oceanospirillales* | *Halomonadaceae* | *Halomonas* |
| 1296 | 5 | 1080 | *Proteobacteria* | *Gammaproteobacteria* | *Vibrionales* | *Pseudoalteromonadaceae* | *Pseudoalteromonas* |
| 1363 | 1 | 2 | *Proteobacteria* | *Gammaproteobacteria* |  |  |  |
| 1387 | 6 | 21 | *Proteobacteria* | *Alphaproteobacteria* | *Sphingomonadales* | *Sphingomonadaceae* | *Sphingomonas* |
| 1427 | 1 | 8 | *Proteobacteria* | *Gammaproteobacteria* | *Oceanospirillales* | *Oceanospirillaceae* |  |
| 1460 | 1 | 1 | *Bacteroidetes* | *Flavobacteriia* | *Flavobacteriales* | *Flavobacteriaceae* | *Flavobacterium* |
| 1540 | 2 | 1 | *Proteobacteria* | *Deltaproteobacteria* | *Myxococcales* |  |  |
| 1605 | 6 | 18 | *Proteobacteria* | *Gammaproteobacteria* | *Alteromonadales* | *Colwelliaceae* | *Thalassomonas* |
| 1776 | 0 | 15 | GN02 | BD1-5 |  |  |  |
| 1922 | 16 | 14 | *Proteobacteria* | *Gammaproteobacteria* | *Oceanospirillales* | *Oceanospirillaceae* | *Marinomonas* |
| 1929 | 0 | 13 | *Proteobacteria* | *Alphaproteobacteria* | *Rhodobacterales* | *Rhodobacteraceae* |  |
| 1981 | 11 | 445 | *Proteobacteria* | *Gammaproteobacteria* | *Oceanospirillales* | *Oceanospirillaceae* | *Marinomonas* |
| 2072 | 1 | 2 | *Proteobacteria* | *Betaproteobacteria* | *Burkholderiales* | *Comamonadaceae* | *Comamonas* |
| 2103 | 5 | 278 | *Proteobacteria* | *Epsilonproteobacteria* | *Campylobacterales* | *Campylobacteraceae* | *Arcobacter* |
| 2146 | 0 | 13 | *Proteobacteria* | *Epsilonproteobacteria* | *Campylobacterales* | *Campylobacteraceae* | *Arcobacter* |
| 2192 | 10 | 650 | GN02 | BD1-5 |  |  |  |
| 2326 | 2 | 48 | *Proteobacteria* | *Gammaproteobacteria* | *Xanthomonadales* | *Xanthomonadaceae* |  |
| 2365 | 2 | 13 | *Proteobacteria* | *Alphaproteobacteria* | *Rhodobacterales* | *Rhodobacteraceae* |  |
| 2374 | 41 | 5 | *Proteobacteria* | *Alphaproteobacteria* | *Rhodobacterales* | *Rhodobacteraceae* | *Paracoccus* |

Shown are results obtained from non-normalized libraries (absolute OTU abundances across the full dataset), which were used in Venn diagram constructions. Values in columns 2W (rearing-water, 2 DAH) and 2L (larvae, 2 DAH) correspond to the total number of sequence reads assigned to OTUs when replicates of each sample category were pooled. Empty cells correspond to taxonomic ranks at each OTUs could not be readily classified. ^1^Total number of quality-filtered sequence reads obtained per microhabitat (see also Table 1).
