# Supplementary material for: Molecular Taxonomic Profiling of Bacterial Communities in a Gilthead Seabream (Sparus aurata) Hatchery
Source: Front Microbiol. 2017 Feb 14;8:204. doi: 10.3389/fmicb.2017.00204 (PMC5306143; doi:10.3389/fmicb.2017.00204)
Supplement: Table S6 — Bacterial OTUs common to live feed and fish larvae (34 DAH). [file Table6.DOCX]

**Table S6│Bacterial OTUs common to live feed and fish larvae (34 DAH).**

| **OTU ID** | **34W** | **34L** | **(%)** | **AM** | **(%)** | **AN** | **(%)** | **RO** | **(%)** | **Class** | **Family** | **Genus** |
| --- | --- | --- | --- | --- | --- | --- | --- | --- | --- | --- | --- | --- |
| 928 | 0 | 1724 | 14,961 | 258 | 2,207 | 0 | 0 | 0 | 0 | *Betaproteobacteria* | *Oxalobacteraceae* |  |
| 2374 | 27 | 736 | 6,387 | 8243 | 70,51 | 1543 | 15,464 | 43 | 0,328 | *Alphaproteobacteria* | *Rhodobacteraceae* | *Paracoccus* |
| 705 | 0 | 661 | 5,736 | 2 | 0,017 | 5 | 0,050 | 0 | 0 | *Gammaproteobacteria* | *Pseudomonadaceae* | *Pseudomonas* |
| 1970 | 0 | 513 | 4,452 | 1 | 0,009 | 2 | 0,020 | 1 | 0,008 | *Alphaproteobacteria* | *Acetobacteraceae* | *Acidocella* |
| 84 | 0 | 280 | 2,429 | 3 | 0,026 | 2 | 0,020 | 0 | 0 | *Gammaproteobacteria* | *Enterobacteriaceae* |  |
| 1953 | 0 | 195 | 1,692 | 0 | 0 | 24 | 0,241 | 0 | 0 | *Gammaproteobacteria* | *Pseudomonadaceae* | *Pseudomonas* |
| 1290 | 0 | 171 | 1,484 | 2 | 0,017 | 123 | 1,233 | 0 | 0 | *Gammaproteobacteria* | *Enterobacteriaceae* | *Klebsiella* |
| 1387 | 0 | 107 | 0,928 | 0 | 0 | 1 | 0,010 | 0 | 0 | *Alphaproteobacteria* | *Sphingomonadaceae* | *Sphingomonas* |
| 807 | 0 | 78 | 0,676 | 0 | 0 | 0 | 0 | 1 | 0,008 | *Alphaproteobacteria* | *Bradyrhizobiaceae* | *Bradyrhizobium* |
| 781 | 9 | 15 | 0,130 | 13 | 0,111 | 238 | 2,385 | 10 | 0,076 | *Gammaproteobacteria* | *Pseudoalteromonadaceae* |  |
| 1262 | 4 | 12 | 0,104 | 13 | 0,111 | 3 | 0,030 | 5 | 0,038 | *Alphaproteobacteria* | *Rhodobacteraceae* |  |
| 1929 | 4 | 12 | 0,104 | 14 | 0,120 | 20 | 0,200 | 16 | 0,122 | *Alphaproteobacteria* | *Rhodobacteraceae* |  |
| 2210 | 0 | 12 | 0,104 | 4 | 0,034 | 1 | 0,010 | 0 | 0 | *Planctomycetia* | *Pirellulaceae* |  |
| 2212 | 0 | 12 | 0,104 | 2 | 0,017 | 6 | 0,060 | 0 | 0 | *Gammaproteobacteria* | *Xanthomonadaceae* | *Stenotrophomonas* |
| 85 | 101 | 11 | 0,095 | 101 | 0,864 | 473 | 4,740 | 72 | 0,550 | *Alphaproteobacteria* | *Rhodobacteraceae* |  |
| 912 | 0 | 10 | 0,086 | 54 | 0,462 | 0 | 0 | 0 | 0 | *Bacilli* | *Carnobacteriaceae* |  |
| 783 | 0 | 9 | 0,078 | 4 | 0,034 | 5 | 0,050 | 0 | 0 | *Bacilli* | *Listeriaceae* | *Brochothrix* |
| 613 | 3 | 8 | 0,069 | 26 | 0,222 | 4 | 0,040 | 16 | 0,122 | *Alphaproteobacteria* | *Hyphomicrobiaceae* |  |
| 708 | 50 | 8 | 0,069 | 1292 | 11,05 | 566 | 5,672 | 184 | 1,405 | *Alphaproteobacteria* | *Rhodobacteraceae* |  |
| 2009 | 5 | 8 | 0,069 | 8 | 0,068 | 11 | 0,110 | 0 | 0 | *Alphaproteobacteria* | *Rhodobacteraceae* |  |
| 569 | 0 | 7 | 0,060 | 0 | 0 | 0 | 0 | 1 | 0,008 | *Planctomycetia* | *Pirellulaceae* |  |
| 1100 | 1 | 6 | 0,052 | 6 | 0,051 | 0 | 0 | 0 | 0 | *Alphaproteobacteria* | *Phyllobacteriaceae* |  |
| 2072 | 0 | 5 | 0,043 | 5 | 0,043 | 0 | 0 | 0 | 0 | *Betaproteobacteria* | *Comamonadaceae* | *Comamonas* |
| 1762 | 8 | 4 | 0,034 | 1 | 0,009 | 1 | 0,010 | 0 | 0 | *Deltaproteobacteria* |  |  |
| 262 | 4 | 3 | 0,026 | 6 | 0,051 | 49 | 0,491 | 6 | 0,046 | *Alphaproteobacteria* | *Hyphomicrobiaceae* | *Devosia* |
| 1369 | 0 | 3 | 0,026 | 0 | 0 | 2 | 0,020 | 0 | 0 | *Gammaproteobacteria* | *Pseudomonadaceae* | *Pseudomonas* |
| 473 | 0 | 2 | 0,017 | 156 | 1,334 | 6 | 0,0601 | 0 | 0 | *Flavobacteriia* | *Flavobacteriaceae* |  |
| 627 | 0 | 2 | 0,017 | 0 | 0 | 1 | 0,010 | 0 | 0 | *Alphaproteobacteria* | *Rhizobiaceae* | *Agrobacterium* |
| 1494 | 0 | 2 | 0,017 | 0 | 0 | 0 | 0 | 6 | 0,046 | *Alphaproteobacteria* | *Rhodobacteraceae* |  |
| 159 | 0 | 1 | 0,009 | 0 | 0 | 1 | 0,010 | 0 | 0 | *Gammaproteobacteria* | *Vibrionaceae* | *Photobacterium* |
| 328 | 1 | 1 | 0,009 | 1 | 0,009 | 0 | 0 | 0 | 0 | *Alphaproteobacteria* | *Rhodobacteraceae* |  |
| 747 | 3 | 1 | 0,009 | 2 | 0,017 | 0 | 0 | 0 | 0 | *Alphaproteobacteria* | *Rhodobacteraceae* | *Phaeobacter* |
| 777 | 0 | 1 | 0,009 | 1 | 0,009 | 0 | 0 | 0 | 0 | *Gammaproteobacteria* | *Pseudomonadaceae* | *Pseudomonas* |
| 861 | 0 | 1 | 0,009 | 28 | 0,240 | 0 | 0 | 0 | 0 | *Bacilli* | *[Exiguobacteraceae]* | *Exiguobacterium* |
| 1432 | 0 | 1 | 0,009 | 4 | 0,034 | 0 | 0 | 0 | 0 | *Alphaproteobacteria* | *Brucellaceae* | *Pseudochrobactrum* |
| 1559 | 0 | 1 | 0,009 | 11 | 0,094 | 4 | 0,0401 | 9 | 0,069 | *Alphaproteobacteria* | *Rhodobacteraceae* |  |

Shown are results obtained from absolute OTU abundances across the full dataset (not normalized), which were used in Venn diagram constructions. Values correspond to the total number of sequence reads assigned to OTUs when replicates of each microhabitat were pooled. (%) indicates the relative abundance of the listed OTUs in each microhabitat. Listed are only OTUs common to fish larvae (34DAH) and at least one of the live feed items used in larval rearing. Abundance values of these OTUs in rearing-water (34DAH) are shown as reference. Sample labels are as in legend to Table 1.
